# Births and induced abortions among women of Russian, Somali and Kurdish origin, and the general population in Finland –comparison of self-reported and register data

**DOI:** 10.1186/s12884-018-1931-x

**Published:** 2018-07-10

**Authors:** Satu Jokela, Eero Lilja, Tarja I. Kinnunen, Mika Gissler, Anu E. Castaneda, Päivikki Koponen

**Affiliations:** 1 Department of Welfare, National Institute for Health and Welfare, Mannerheimintie 166, 00271 Helsinki, PL 30 Finland; 2 Faculty of Social Sciences, University of Tampere, PL 100, Arvo Ylpön katu 34, Tampere, 33520 Finland; 3 Department of Information Services, National Institute for Health and Welfare, PL 30, Mannerheimintie 166, Helsinki, 00271 Finland; 4 Department of Neurobiology, Care Sciences and Society, Division of Family Medicine, Karolinska Institute, Stockholm, Sweden; 5 Department of Public Health Solutions, National Institute for Health and Welfare, Mannerheimintie 166, 00271 Helsinki, PL 30 Finland

**Keywords:** (5) reproductive health, Migrants, Women, Parturition, Induced abortion

## Abstract

**Background:**

Since reproductive health is often considered a highly sensitive topic, underreporting in surveys and under coverage of register data occurs frequently. This may lead to inaccurate information about the reproductive health. This study compares the proportion of women having births and induced abortions among migrant women of Russian, Somali and Kurdish origin in Finland to women in the general Finnish population and examines the agreement between survey- and register-based data.

**Methods:**

The survey data from the Migrant Health and Wellbeing Study conducted in 2010–2012 and data from the Health 2011 Survey with corresponding information on women in the general population were used in this study. The respondents were women aged 18–64: 341 Russian, 176 Somali and 228 Kurdish origin women and 630 women in the general population. The survey data were linked to the Finnish Medical Birth Register and the Register of Induced Abortions.

**Results:**

In the combined (survey and register) data, migrant groups aged 30–64 had a higher proportion (89–96%) compared to the general population (69%) of women with at least one birth. Under-coverage of registered births was observed in all study groups. Among women aged 18–64, 36% of the Russian group and 24% of the Kurdish group reported more births in the survey than in the register data. In the combined data, the proportions of Russian origin (69%) and Kurdish origin (38%) women who have had at least one induced abortion in their lifetime are higher than in the general population (21%). Under-reporting of induced abortions in survey was observed among Somali origin women aged 18–29 (1% vs. 18%). The level of agreement between survey and register data was the lowest for induced abortions among the Somali and Russian groups (− 0.01 and 0.27).

**Conclusion:**

Both survey- and register-based information are needed in studies on reproductive health, especially when comparing women with foreign origin with women in the general population. Culturally sensitive survey protocols need to be developed to reduce reporting bias.

## Background

Reproductive health is an important component of general health especially among women [1, 2]. It deals with the reproductive processes, functions and system at all stages of women’s life [3]. Reproductive health and family planning services are available to all women in Finland. Most families are able to plan the number of children they desire as reliable methods of contraception are available to virtually everyone [4]. However, it has been found that it is more likely for women with only a basic education to have an abortion than it is for women with more education [5].

In 2015, the total fertility rate (births per women) in Finland was 1.65 [6, 7] and the number of induced abortions per thousand women aged 15–49 was 8.2 (13.3. in other Nordic countries). The number of induced abortions has decreased especially among women aged less than 20 years in all Nordic countries, being the lowest in Norway and in Finland [8]. The number of induced abortions among the other European countries was the highest in the area of former Soviet Union [6].

Some groups of migrant women and especially refugee women do not have equal access to health services nor access to reproductive health information neither in their home country nor their host country. Previous studies [9–12] have showed barriers to the use of health care services resulting from gender-related issues (such as patrilineal, patrilocal family systems), traditional values, norms and beliefs (such as perceptions of femininity, health beliefs and stigmatization), discrimination among health care providers, limited services for adolescents and unmarried women, as well as lack of adequate knowledge and information on sexual and reproductive health.

In Finland, a register-based study showed that the proportion of immigrant women among the women having an induced abortion had slightly increased between 1994 (4.6%) and 2002 (7.8%). The abortion rate increased particularly among young women of Somali and other African origin. The abortion rate among women of non-Western origin was lower than among Finnish women. Baltic, Chinese, Russian, Thai & Filipina, and African women aged 15–49 had more abortions per 1000 women than Finnish women in the same age group [13].

Also other European register-based studies [14, 15] and surveys [16, 17] show that abortion rates are higher among migrant women than in the general population and migrant women request abortion more often than women in the general population [14, 16]. Abortions have found to be associated with low education, weak social network, poverty, unemployment, and having limited access to healthcare [15, 16]. Immigrant women may thus be more vulnerable to abortions than women in the general population [15, 17].

Under-reporting of induced abortions in surveys is a generally recognized problem [18]. The usefulness of surveys in studying highly personal or sensitive individual characteristics, such as sexual reproductive health, has been questioned [19], but the usefulness of a survey depends on cultural issues as well as the data collection methods used. A study in the United States found that only 29% of actual induced abortions shown in Medicaid administrative data files were self-reported in a survey. The level of underreporting varied by ethnicity: white women reported about 71%, black women reported 24% and Hispanic women 34% of their actual abortions [20]. Under-reporting of induced abortions has been associated with socio-demographic characters, experiences in life (e.g. reproductive history) and the women’s own and general attitudes on abortions and the context of the survey (for example the sex and training of interviewers) [20–25].

In this cross-sectional study we have examined the agreement between survey- and register –based data in comparing the proportions of women having births and induced abortions among migrant women of Russian, Somali and Kurdish origin to women in the general Finnish population. Previously very few studies have utilized both survey- and register data [18, 20, 21, 25].

The results can be utilized to improve estimates of fertility and unintended pregnancies, as well as to evaluate needs to improve access to services and use of contraceptives [18, 20, 24, 26].

## Methods

### Study population

The data on Russian, Somali and Kurdish origin migrant women living in Finland are from the Migrant Health and Wellbeing Study (Maamu) conducted by the National Institute of Health and Welfare (THL) between 2010 and 2012. The Maamu study is a comprehensive cross-sectional health interview and examination survey. The three groups of origin were selected to represent different types of migrants. Russian migrants are the largest migrant group in Finland with family and work as their main reasons for migration. Somali migrants are the largest refugee group, whereas Kurdish migrants coming from Iran or Iraq have been among the largest groups of quota refugees over the past decade. The survey data was collected by trained multilingual personnel. The study protocol included a face-to-face interview on health and wellbeing and a health examination [27]. A stratified random sample of 1000 Russian (622 women), 1000 Somali (531 women), and 1000 Kurdish (426 women) origin adults aged 18 to 64 and living in six Finnish municipalities (Helsinki, Espoo, Vantaa, Turku, Tampere and Vaasa) was selected from the National Population Register. Selection criteria for Russians were birthplace in the Former Soviet Union or Russia and mother tongue Russian or Finnish, for Somalis birthplace in Somalia, and for Kurdish birthplace in Iraq or Iran and mother tongue Kurdish Sorani. The invitees had a minimum one year of residence in Finland. In this study, we used the data that included the women who had participated in the interview. A total of 54.8% of invited Russian (*n* = 341), 36.0% of Somali (*n* = 191) and 54.0% of Kurdish (*n* = 230) origin women participated in the face-to-face interviews and answered questions on reproductive health.

The comparison group of the general Finnish population women is from the data of nationwide study, Health 2011 Survey. The study was conducted by THL in 2011–2012 [28]. Women aged between 18 to 64 and living in the same municipalities as in the Maamu study were included in the general population group (*n* = 630). The response rate in Health 2011 was 53%.

Both Maamu and Health 2011 surveys were approved by the Coordinating Ethical Committee of Helsinki and Uusimaa Hospital District. All participants gave written informed consent.

### Data sources

Two data sets have been used in this study: 1) the survey data from two surveys: the Maamu and Health 2011 and 2) the register data from two registers: the Finnish Medical Birth Register (MBR) and the Register of Induced Abortions. Furthermore, we created combined data from these data sets by linking the self-reported survey data to the register data [8, 29].

The MBR contains data on all live births and stillbirths that have occurred in Finland since 1987. Furthermore the MBR includes information on the number of previous pregnancies, births and induced abortions that have been self-reported in maternity health services. If a woman has ever given birth in Finland, the MBR will also contain data on self-reported births and induced abortions in the woman’s lifetime, including those that had taken place before the register was set up and those that had been performed in other countries [29].

The Register of Induced Abortions contains information on all legally induced abortions in Finland. The register also includes information on the number of previous abortions that have been self-reported when seeking abortion/having the abortion as well as the number of previous pregnancies reported while in maternity care. The physician performing an abortion is required to report the case to THL. Data on induced abortions from 1983 onwards are available in electronic format [8]. In this study, the register data were limited to the time period before the person’s participation in the interview. The coverage and validity of data on induced abortions in the Register of Induced Abortions [30] and on births in the MBR register [31] are good. Register linkages were made using the unique personal identity code given for all citizens and permanent residents living in Finland. Therefore it is possible to compare individual women’s births and induced abortions between survey and register datasets (Table 1).

**Table 1:** Information on previous births and induced abortions in three data sets

The Maamu survey data	The Finnish Medical Birth Register	The Register of Induced Abortions
Self-reported births	Registered births (from year 1987)	Registered induced abortions (from year 1983)
Self-reported induced abortions	Self-reported previous births among those with given births in Finland since 1987	Self-reported previous induced abortions among those with induced abortions in Finland since 1983
	Self-reported previous induced abortions among those with given births in Finland since 1987	Self-reported previous births among those with induced abortions in Finland since 1983
Combined survey and register data		
maximum number of births/induced abortions in survey or register		
dichotomous variables for births/induced abortions: 1) no births/induced abortion, 2) at least one birth/induced abortion		
categorized number of births: no births, 1–2 births and 3 or more births		
categorized number of induced abortion: no induced abortions, one induced abortion and two or more induced abortions		

### Definition of variables

In the Maamu study, the interviews were conducted in the native language of the participants mainly by female interviewers. The issue of births were addressed with the following question: “How many births have you had? Include all births, also Caesarean sections.” The issue of induced abortions were addressed with following question: “Have you had any induced abortions and how many?” In the Health 2011 survey, questions on reproductive health were included in the interview for persons aged 29 to 64 while for young adults (aged 18 to 28) these questions were included in a self-administered postal questionnaire.

We found differences between the self-reported data and the register data regarding the amount of births and induced abortions. We created a variable on the maximum values of births/induced abortions by choosing whichever was the highest value, self-reported or registered. These continuous variables were used for mean numbers of births and induced abortions.

Because of the observed differences between data sources, we also created dichotomous variables for births and induced abortions: 1) no births and 2) at least one birth and 1) no induced abortions and 2) at least one induced abortion. We also created variables on the number of births and induced abortions with three categories (no births, 1–2 births and 3 or more births and no induced abortions, one induced abortion and two or more induced abortions).

In order to highlight the differences between the age groups, the results of women aged 18–29 and 30–64 will be discussed separately.

We also present mean numbers of births among parous women and among all women and mean numbers of induced abortions among women who have had at least one induced abortion and among all women. Furthermore we present proportions of having 1–2 births and more than two births and mean number of births in lifetime among women aged 18–64 who have had their first birth after migration.

We used the aforementioned categorical variables when comparing the information from the survey data and the register data (more births/induced abortions in survey, similar reporting in survey and register data and more births/induced abortions in register data) and when examining the level of agreement between register- and survey- based data.

### Statistical analyses

Inverse probability weights (IPW) based on the register information from the National Population Register on age, sex, marital status, migrant group and municipality were used to correct for the effects of non-response bias and different sampling probabilities in order to provide representative results with the survey and register data [32]. The population size being relatively small, a significant proportion of the total population was included in the sample of Maamu, and thus the finite population correction [33] was applied in all analyses.

Linear regression was applied to calculate age adjusted mean values for births and induced abortions. Logistic regression was applied to calculate model- adjusted estimates for having births or induced abortion and their 95% confidence interval (CI) for binary and multinomial variables. First age-adjusted and second age-, employment status-, marital status- and education adjusted proportions of women having births and induced abortions were estimated in each study group using predicted margins [34]. Weighted Cohen’s kappa was used to examine the level of agreement between register and survey based data. The statistic was calculated for the three-category variables of births and induced abortions in each group [35]. The statistical significances of the differences between groups were calculated using Satterthwaite adjusted F-statistic. A *p*-value of < 0.05 was considered statistically significant. SAS software (9.3) was used for constructing outcome variables and calculating crude values, whereas SUDAAN 11.0.1 was used for data analysis.

## Results

### Characteristics of the study population

The main characteristics of the women who answered the questions considering reproductive health are presented in Table 2. Somali and Kurdish women were younger than Russian and general population women. Most of the women were married or lived in a civil union. The proportion of women who had no formal education was highest in the Somali group (35%). Most migrant women had lived in Finland for at least five years. In the general population, more women (84%) were employed than in the Somali (37%), Kurdish (56%) and Russian (66%) groups.

**Table 2:** Characteristics of the study population

	General population (*n* = 630) %, 95 CI	Russian (*n* = 341) %, 95 CI	Somali (*n* = 176) %, 95 CI	Kurdish (*n* = 228) %, 95 CI
Marital status				
Unmarried/divorced/widow	39.1 (35.2–43.1)	43.6 (37.8–49.6)	32.3 (25.5–39.9)	33.7 (28.0–39.8)
Married/civil union	60.9 (56.9–64.8)	56.4 (50.4–62.2)	67.7 (60.1–74.5)	66.3 (60.2–72.0)
Education				
No formal education	0.0 (NA)	0.0 (NA)	35.3 (28.4–43.0)	19.4 (15.1–24.7)
Primary	27.7 (24.1–31.5)	15.5 (11.7–20.3)***	49.3 (41.4–57.3) ***	39.3 (33.5–45.5) ***
Secondary/higher	72.3 (68.5–75.9)	84.5 (97.7–88.3) ***	15.4 (10.6–21.8) ***	41.2 (35.4–47.3) ***
Employment status				
Working	83.7 (80.4–86.6)	65.8 (60.2–71.0) ***	37.3 (30.3–44.9) ***	56.2 (50.2–62.1) ***
Others	16.3 (13.4–19.6)	34.2 (29.0–39.8) ***	62.7 (55.1–69.7) ***	43.8 (37.9–49.8) ***
Moved to Finland (age yrs)				
≤18 yrs		38.4 (32.8–44.4)	46.2 (38.1–54.5)	41.4(34.9–48.2)
>18 yrs		61.6 (55.6–67.2)	53.8 (45.5–61.9)	58.6 (51.8–65.1)
Time lived in Finland				
≤5 yrs		23.0 (18.4–28.5)	20.6 (15.5–26.7)	16.6 (12.7–21.3)
6–14 yrs		37.6 (32.1–43.4)	38.9 (31.5–46.8)	56.0 (49.9–61.9)
≥15 yrs		39.4 (33.7–45.4)	40.6 (33.2–48.4)	27.5 (22.4–33.2)

**Survey data**:

Migrant Health and Wellbeing Study and the Health 2011 Survey

Statistically significant differences compared to the Finnish reference group (Satterthwaite adjusted F-statistic):

*p-value < 0.05

**p-value < 0.01

***p-value < 0.001

Age-adjusted

NA = Confidence interval not available

### Births

In the combined data Somali and Kurdish origin women aged 30–64 had more often had at least one birth in their lifetime (96%) than the other groups. (Table 3) In the general population, of women aged 18–29 9 % had given birth at least once in their lifetime. All migrant groups had significantly higher proportions of women aged 30–64 with at least one birth compared to the general population women. Somali origin women who had had their first birth before the migration were more likely to have three or more births compared to those who had had their first birth after the migration (91 and 69%).

**Table 3:** The distribution of births in different data sets

	General population	Russian	Somali	Kurdish
18–29 years ^a^	*n* = 182	*n* = 83	*n* = 70	*n* = 66
Survey data ^1^, women with at least one birth, %	8.5 (5.1–13.8)	17.0 (11.0–25.3)*	60.3 (48.1–71.3)***	41.2 (33.1–49.8)***
Register data ^2^, women with at least one birth, %	8.5 (5.1–13.8)	16.1 (10.3–24.4)*	54.5 (42.8–65.7)***	37.6 (30.0–46.0)***
Combined data ^3^, women with at least one birth, %	8.5 (5.1–13.8)	17.0 (11.0–25.3)*	60.3 (48.1–71.3)***	41.2 (33.1–49.8)***
30–64 years ^b^	*n* = 448	*n* = 258	*n* = 104	*n* = 161
Survey data ^1^, women with at least one birth, %	69.2 (63.9–74.0)	89.0 (84.0–92.5)***	95.5 (89.9–98.1)***	95.7 (91.7–97.9)***
Register data ^2^, women with at least one birth, %	52.9 (47.6–58.1)	44.7 (38.4–51.1)*	67.5 (58.2–75.6)**	62.3 (55.3–68.8)*
Combined data ^3^, women with at least one birth, %	69.2 (63.9–74.1)	89.0 (84.0–92.5)***	95.5 (89.9–98.1)***	96.4 (92.6–98.3)***
18–64 years ^c^, Combined data ^3^	*n* = 630	*n* = 341	*n* = 174	*n* = 227
Among all women				
no births, %	50.6 (46.9–54.2)	33.5 (29.0–38.3)***^5^	17.6 (13.1–23.3)***^5^	21.8 (17.8–26.5) ***^5^
1–2 births, %	36.8 (33.2–40.5)	58.6 (53.5–63.5)	18.5 (13.3–25.0)	36.4 (30.8–42.4)
≥3 births, %	12.6 (10.3–15.4)	7.9 (5.6–11.2)	63.9 (57.4–70.0)	41.8 (36.7–47.0)
Mean number of births among all women	1.0 (1.0–1.1)	1.0 (0.9–1.1)	4.5 (4.0–5.0)***	2.6 (2.3–2.8)***
Mean number of births among parous women	1.8 (1.7–2.0)	1.6 (1.4–1.7)**	5.9 (5.3–6.4)***	3.4 (3.1–3.7)***
Among women with first birth before migration		*n* = 179	*n* = 57	*n* = 117
1–2 births, %	NA	86.2 (80.2–90.6)	8.7 (3.7–19.3)	32.0 (24.5–40.5)
≥3 births, %	NA	13.8 (9.4–19.8)	91.3 (80.7–96.3)	68.0 (59.5–75.5)
mean	NA	1.6 (1.4–1.7)	7.2 (6.4–8.1)	3.8 (3.5–4.2)
Among women with first birth after migration †		*n* = 72	*n* = 50	*n* = 65
1–2 births, %	NA	92.7 (81.6–97.3)	30.8 (18.3–46.8)***	72.9 (60.1–82.7)***
≥3 births, %	NA	7.3 (2.7–18.4)	69.2 (53.2–81.7)***	27.1 (17.3–39.9)***
mean	NA	1.7 (1.5–2.0)	4.2 (3.6–4.73)***	2.4 (2.1–2.7)***
Women with at least one birth, model-adjusted ^d %^	61.0 (56.3–65.6)	76.4 (72.0–80.3)***	85.7 (80.8–89.5)***	83.9 (80.2–87.1)***

^1^**Survey data**:

Migrant Health and Wellbeing Study and the Health 2011 Survey

Age-adjusted and weighted proportions

^a^18–29 years age adjusted

^b^30–64 years age adjusted

^2^ **Register data:**

Medical Birth Register and the Register of Induced Abortions

Age-adjusted and weighted proportions

^3^ **Combined survey and register data:**

Combined data from the surveys, Medical Birth Register and Register of Induced Abortions

Age-adjusted and weighted proportions

^c^18–64 years age adjusted

† Statistical differences in relation to migrant origin women whose first birth was before migration

NA = no observations

^d^Adjusted for age, work status, marital status and education

Statistically significant differences compared to the Finnish reference group (Satterthwaite adjusted F-statistic):* *p*-value < 0.05

**p-value < 0.01

***p-value < 0.001

^5^Overall *p*-value for the difference of the multinomial distribution between general population and the migrant group

The mean number of births was highest among Somali origin women; 5.9 among parous women aged 18–64 and 4.5 among all women aged 18–64. When adjusting for age, employment status, marital status and education there was still a significant difference between the proportion of women having at least one birth in Russian, Somali and Kurdish origin women and women in the general population.

### Induced abortions

Based on the survey data, 1 % of Somali origin women aged 18–29 had gone through at least one induced abortion in their lifetime (Table 4), while based on the register data the proportion was much higher (18%). Instead, 66% of Russian origin women aged 30–64 had had at least one induced abortion in survey data but only 23% had had at least one induced abortion based on the register data.

**Table 4:** The distribution of induced abortions in different data sets

	General population	Russian	Somali	Kurdish
18–29 years ^a^	*n* = 181	*n* = 83	n = 65	n = 63
Survey data ^1^, women with at least one induced abortion, %	6.6 (3.6–11.8)	19.6 (11.4–31.5)**	0.7 (0.1–3.9)*	3.6 (1.0–11.8)
Register data ^2^, women with at least one induced abortion, %	7.0 (3.9–12.2)	12.5 (6.1–23.8)	17.6 (9.0–31.5)*	6.2 (2.6–14.2)
Combined data ^3^, women with at least one induced abortion %	7.7 (4.5–13.1)	20.0 (11.8–31.9)**	18.5 (9.8–32.1)*	7.8 (3.6–15.9)
30–64 years ^b^	*n* = 447	*n* = 255	*n* = 99	*n* = 159
Survey data ^1^, women with at least one induced abortion, %	16.4 (13.1–20.3)	66.2 (59.7–72.1)***	1.1 (0.4–3.2)***	31.2 (24.6–38.6)***
Register data ^2^, women with at least one induced abortion, %	17.7 (14.2–21.8)	22.8 (17.6–29.1)	7.6 (3.6–15.0)*	21.8 (16.7–28.0)
Combined data ^3^, women with at least one induced abortion, %	20.8 (17.2–25.0)	68.9 (62.5–74.7)***	10.1 (5.2–18.7)*	37.8 (30.9–45.3)***
18–64 years ^c^, combined data ^3^	*n* = 682	*n* = 308	*n* = 164	*n* = 222
no induced abortions, %	83.2 (80.0–85.9)	46.6 (41.2–52.0)***^5^	85.6 (77.6–91.1)	71.8 (66.2–76.9)***^5^
one induced abortion, %	13.5 (11.1–16.6)	21.3 (16.8–26.5)	8.4 (4.6–14.9)	14.9 (11.1–19.8)
≥2 induced abortions, %	3.3 (2.2–5.0)	32.2 (27.6–37.1)	6.0 (2.7–12.8)	13.2 (9.6–18.0)
Mean number of abortions				
among women who had at least one induced abortion	1.3 (1.1–1.4)	2.3 (2.1–2.6) ***	2.0(1.5–2.5)**	2.0 (1.6–2.2)***
among all women	0.2 (0.2–0.3)	1.3 (1.1–1.5)***	0.3 (0.2–0.4)	0.5 (0.4–0.7)***
Women with at least one induced abortion, model-adjusted ^d^ %	17.9 (14.7–21.6)	60.0 (54.4–65.4)***	NA	35.3 (28.8–42.5)***

^1^ **Survey data:**

Migrant Health and Wellbeing Study and the Health 2011 Survey

Age-adjusted and weighted proportions

^a^18–29 years age adjusted

^b^30–64 years age adjusted

^2^ **Register data:**

Medical Birth Register and the Register of Induced Abortions

Age-adjusted and weighted proportions

^3^ **Combined survey and register data:**

Combined data from the surveys, Medical Birth Register and Register of Induced Abortions

Age-adjusted and weighted proportions

^c^18–64 years age adjusted

NA = Too few observations for statistical analysis

^d^Adjusted for age, work status, marital status and education

Statistically significant differences compared to the Finnish reference group (Satterthwaite adjusted F-statistic):

*p-value < 0.05

**p-value < 0.01

***p-value < 0.001

^5^Overall *p*-value for the difference of the multinomial distribution between general population and the migrant group

In the combined data, higher proportions of Somali origin women aged 18–29 had had at least one induced abortion than women in the general population (19 and 8%) whereas a higher proportion of general population women aged 30–64 had had at least one induced abortion compared to Somali origin women (21 and 10%). Russian and Kurdish origin women aged 30–64 had higher proportions of those with at least one induced abortion than general population women in the same age group. The mean number of induced abortions was the highest among Russian origin women; 2.3 among women with at least one induced abortion and 1.3 among all women.

When adjusting for age, employment status, marital status and education, significantly lower proportions of general population women had had induced abortions (18%) compared to Russian (60%) and Kurdish (35%) origin women. The number of Somali origin women who had had an induced abortion was too low to be adjusted for sociodemographic factors.

### Comparison between data sets

When comparing the self-reported survey information to the register data, all study groups showed more births in the survey data than in the register data (Table 5). Among women aged 18–64, 36% of the Russian group and 24% of the Kurdish group reported more births in the survey compared to the register data. The level of agreement between self-reported survey information and register data for births was moderate or substantial among all women (kappa 0.46–0.74).

**Table 5:** Agreement between data sets: proportions of women (%) and Cohen’s kappa values

	General population	Russian	Somali	Kurdish
Births				
more self-reported in survey, %	14.0 (11.6–16.8)	36.1 (30.7–41.9)	16.3 (11.7–22.3)	24.2 (19.4–29.7)
similar, %	85.3 (82.5–87.7)	63.8 (58.0–69.2)	82.9 (76.8–87.7)	74.9 (69.3–79.7)
more in register, %	0.7 (0.3–1.7)	0.0 (NA)	0.8 (NA)	0.9 (NA)
Cohen’s kappa	0.75	0.46	0.69	0.56
Induced abortions				
more self-reported in survey, %	3.6 (2.4–5.3)	38.6 (33.0–44.4)	0.9 (0.4–2.1)	8.3 (5.4–12.5)
similar, %	92.3 (90.0–94.1)	58.5 (52.6–64.1)	87.2 (80.1–92.0)	83.9 (78.8–88.0)
more in register, %	4.1 (2.9–5.8)	3.0 (1.6–5.6)	11.9 (7.2–19.1)	7.8 (5.1–11.9)
Cohen’s kappa	0.69	0.27	−0.01	0.56

Combined data from Maamu, Health 2011, MBR and Register of Induced Abortions

NA = Confidence interval not available

Only 1 % of Somali origin women reported more induced abortions in the survey than in the register data, while 12% of them had more induced abortions in the register data. In contrast, Russian origin women had more self-reported previous induced abortions than appeared in the register data (39 and 3%). Among Somali origin women, the level of agreement between self-reported survey information and register data for induced abortions was lower than the expected probability of agreement at random (kappa − 0.01) and among Russian origin women agreement was fair (kappa 0.27). The level of agreement was moderate among the Kurdish (0.56) and the general population (0.69) groups.

## Discussion

### Births and induced abortions

We compared the proportions of women having births and induced abortions among migrant women of Russian, Somali and Kurdish origin to women in the general Finnish population. In our study, Somali origin women had on average more births than the other groups. In the combined data, 96% of Somali origin women had had three or more births. The mean number of births among parous Somali origin women aged 18–64 was 5.9. Adjusting for age, employment status, marital status and education did not much impact the differences between our study groups. Even when adjusted for confounders migrant origin women still had significantly more often had at least one birth compared to the women in the general population.

Russian origin women had on average more induced abortions than the other groups. In the combined data, 32% of Russian origin women had had two or more induced abortions. The mean number of induced abortions among Russian origin women who had had at least one induced abortion was 2.3. Furthermore, when adjusted for confounders, Russian and Kurdish origin women had significantly more often had an induced abortion when compared to the general population women. Although the numbers of respondents to the question about induced abortions are fairly low, the differences between migrant origin women and general population women are clearly significant.

There are many explanations for the differences in the proportions and mean numbers of births and induced abortions between migrant groups and women in the general population. Due to the heterogeneity of migrant women, there is no reason to assume that higher proportions of induced abortion in some groups of women are only due to their cultural background [16] or lower education. Previous studies have shown that there are many reasons for induced abortion, such as being a single mother, already having children and young age [17] as well as other reasons that are related to low socio-economic status. Also women with good socio-economic situation undergo induced abortions [36].

A previous study has shown that Russian origin women and the oldest Russian speaking Estonians had higher levels of self-reported induced abortions compared to other Estonian and Finnish women. High abortion rates were related to low contraceptive use [37].

### Agreement between survey- and register data

We compared survey-based and register-based information to examine the level of agreement between the data sets. In this study, under-coverage of registered births was observed in all study groups. Among women aged 18–64, 36% of the Russian group and 24% of the Kurdish group reported more births in the survey compared to the register data. Somali origin women aged 18–29 under-reported induced abortions in survey compared to register data (1% vs. 18%). The level of agreement between survey and register data was lowest for induced abortions among the Somali and Russian groups.

In the general population women, the under-coverage of registered births is partly due to the births before year 1987 when the MBR was not in use. Furthermore, some general population women probably have given birth abroad. Most of the migrant women who reported more births in the survey have probably given birth elsewhere.

We observed substantial differences in the proportions of induced abortions between the two data sources. Especially Somali origin women under-reported their previous induced abortions, whereas Russian women had more self-reported abortions in the survey data. It is likely that the Russian women in our study had had induced abortions in Russia before they have moved to Finland or they might have travelled to Russia to get an induced abortion, leading to under-coverage of the register data.

The level of agreement between register- and survey-based data on induced abortions was the lowest among Somali origin women. Somali origin women might under-report induced abortions because termination of pregnancy is not culturally acceptable and there is a high level of social stigma related to induced abortions [18].The observed differences between self-reported and registered previous induced abortions among Somali and Kurdish origin women might be partly caused due to problems in the data collection process as well as the sensitive nature of these questions.

### Meaning of the results

Our study clearly demonstrates the need for taking action in order to promote migrant women’s reproductive health especially in family planning and abortion services. The high induced abortion levels among Russian origin women show a need to pay attention to the availability of contraceptives and family planning services. The high mean number of births among Somali origin women may reflect their personal wishes for larger families but there is also a need to make sure that there are no unnecessary barriers to use contraceptives, eg. their husbands’ or their own beliefs and attitudes [38, 39].

Somali origin women who had had their first birth after migration, had less births, compared to Somali origin women who had had their first birth before moving to Finland. Part of the explanation might be that women who had given birth after migration where younger, but it might also reflect the fact that their fertility has decreased closer to the fertility of the majority of the population [40].

In order to maintain good reproductive health, women need to have access to information, prevention and treatment services [41]. Sexual education contributes to the use of contraception [42] but it should be noted that birth control is a complicated question in terms of cultural, religious and social norms [43] and therefore particular attention must be paid to women’s ability to make their own decisions considering their sexual and reproductive health.

Health care professionals need more information on how to better take into account migrant origin women’s special needs (such as information on family planning and trustable contraceptive methods). Depending on the country of origin, migrant women might also need information on women’s rights and gender equality to improve their access and use of sexual and reproductive health care services [44, 45].

### Strengths and limitations

The strength of our study is that it is based on a population-based survey among three major migrant groups in Finland. Moreover, Finland’s population-based health registers have a high coverage of births and induced abortions in case a woman has given birth or had an induced abortion in Finland [30, 31]. The Health 2011 and Maamu -surveys had a relatively high participation rate, compared to most previous surveys among general populations [46], but especially when compared to response rates among migrant populations [47, 48].

Other strengths of this study are that the survey data were collected by trained fieldwork staff using standard protocols and questions to ensure comparability between the study groups. However, younger participants in the general population received the questionnaire by post and this may have affected their responses when compared to the face-to-face interviews.

All analyses were conducted using inverse probability weights to correct for the effects of non-response and different sampling probabilities. However, the small numbers of participants and selective non-response can cause some bias, especially in the Somali group which has the lowest response rate and low numbers of women reporting induced abortions.

In the Maamu study, the aim was that the questions on women’s reproductive health would be asked by female interviewers with the same origin as the respondent whenever it was possible. It was assumed that sensitive questions (such as contraceptive use, births, miscarriages and abortions) were more culturally acceptable when asked by a same-sex interviewer. Unfortunately, e.g. due to absences of multilingual interviewers, these questions were in a few occasions asked by male interviewers. As the gender of the interviewer was not systematically recorded, we are not able to analyse whether this had an effect on the reporting.

Item non-response may have caused some bias for Somali and Kurdish origin women. Discussions with the interviewers revealed that a few interviewers had skipped the questions as they thought that it was inappropriate to ask questions about induced abortions and births when the interviewer was male, younger than the interviewee, or if the woman was unmarried. The low level of agreement between survey and register data on induced abortions among Somali origin women might be partly due to these problems in the data collection process. However, it is likely that abortions are underreported because they are not socially approved.

Because of the obvious inaccuracy of the numbers of births and induced abortions both self-reported and registered, we couldn’t compare the exact numbers of births and induced abortions in the two data sets. Instead of examining the exact validity, we compared the agreement between these dataset using categorical variables.

Unfortunately, the number of women who had lived in Finland their whole reproductive age was low (less than 50 in each migrant group) and it was not possible to compare reporting of induced abortions and births among women who had moved to Finland when they were younger than 15 years old, compared to women who had moved to Finland when they were older.

## Conclusions

Our study demonstrates that by using both survey- and register-based data it is possible to get more accurate information on women’s reproductive health. Both survey- and register-based information, should be used when a study focuses on sensitive subject areas such as women’s reproductive health, especially induced abortions. Attention should also be given to the training of survey interviewers and the need for same-sex interviewers as well as developing other methods to improve culturally sensitive survey protocols.
